# Amyloid fibrils embodying distinctive yeast prion phenotypes exhibit diverse morphologies

**DOI:** 10.1093/femsyr/foy059

**Published:** 2018-05-25

**Authors:** Rupam Ghosh, Jijun Dong, Joe Wall, Kendra K Frederick

**Affiliations:** 1 Department of Biophysics, University of Texas Southwestern Medical Center, Dallas, TX 75390; 2 Alkermes Inc. 852 Winter Street, Waltham, MA 02451; 3 Brookhaven National Laboratory, Upton, NY 11973

**Keywords:** amyloid, prion, prion strains, Sup35, structural biology, scanning transmission electron microscopy (STEM), solid-state nuclear magnetic resonance (NMR)

## Abstract

Yeast prions are self-templating protein-based mechanisms of inheritance whose conformational changes lead to the acquisition of diverse new phenotypes. The best studied of these is the prion domain (NM) of Sup35, which forms an amyloid that can adopt several distinct conformations (strains) that confer distinct phenotypes when introduced into cells that do not carry the prion. Here, we investigate the structure of NM fibrils templated into the prion conformation with cellular lysates. Our electron microscopy studies reveal that NM fibrils that confer either a strong or a weak prion phenotype are both mixtures of thin and thick fibrils that result from differences in packing of the M domain. Strong NM fibrils have more thin fibrils and weak NM fibrils have more thick fibrils. Interestingly, both mass per length and solid state NMR reveal that the thin and thick fibrils have different underlying molecular structures in the prion strain variants that do not interconvert.

## Teaser

Structural characterization of the different shapes adopted by the yeast prion protein responsible for non-Medialian inheritance in yeast reveals several distinct forms.

## INTRODUCTION

Yeast prions are self-templating, protein-based genetic elements whose conformational changes enable enhanced phenotypic diversity (Shorter and Lindquist 2005). One of the best-studied prions, [*PSI^+^*] (Cox 1965), results from the sequestration of Sup35 in a heritable amyloid state, leading to the reduction of translation termination efficiency (True and Lindquist 2000; True, Berlin and Lindquist 2004). Most prions, including [*PSI^+^*], exhibit stable phenotypic changes of varying magnitudes called ‘strains’ (Prusiner *et al.*^1998^; Chien, Weissman and DePace 2004; Diaz-Avalos *et al.*^2005^; Krishnan and Lindquist 2005). The strength of a strain is determined by the delicate interplay between the thermodynamic stability of the intermolecular interactions between protein monomers and the activity of the prion propagation machinery that fragments the fibers (Diaz-Avalos *et al.*^2005^; Krishnan and Lindquist 2005). The [*PSI^+^*] prion embodies least two, and possibly more, strains (Bateman and Wickner 2013). Strong and weak [*PSI^+^*] strains have different stop-codon read-through phenotypes. Sup35 expression levels are the same in [*psi*^−^] and [*PSI^+^*] cells, as they are in weak and strong [*PSI^+^*] strains. The differences in phenotype are caused by having differing amounts of soluble Sup35 available for translation termination (Uptain *et al.*^2001^), and possibly differences in residual activity of Sup35 monomers in the amyloid fibers (Baxa *et al.*^2011^). Recruitment of monomers into the amyloid fiber is limited not by the rate of templating per se, but by the number of free templating ends available from fiber fragmentation (Cox, Ness and Tuite 2003; Frederick *et al.*^2014^). NM fibers producing strong phenotypes are thermodynamically less stable and more easily fragmented. They recruit a greater fraction of Sup35 monomers into the amyloid form and produce a stronger phenotype. Conversely, NM fibers producing weak phenotypes are more stable, recruit fewer monomers, and leave behind a larger pool of functional Sup35. Non-Mendelian inheritance of specific protein conformations that produces different phenotypes with a genetically identical population indicates the presence of protein-only inheritance.

Structural polymorphisms in the aggregated state of Sup35 amyloids give rise to different phenotypes of yeast that result in the various yeast prion strains (Diaz-Avalos *et al.*^2005^; Krishnan and Lindquist 2005). Biophysical studies reveal that the amyloids of Sup35 that can confer different biological phenotypes have distinct biophysical properties and underlying conformations. Multiple lines of evidence have delineated the roles of the three domains in Sup35 in prion biology. The N-terminal region of Sup35 is responsible for the polymerization reaction of native soluble Sup35 into the amyloid form. This region (residues 1–123 of Sup35) is extremely rich in polar uncharged residues, especially glutamine and asparagine residues (together, 45%) and is involved in prion propagation within the cell. This region includes 5.5 copies of an imperfect oligopeptide repeat with the consensus sequence PQGGYQQ-YN (residues 46 - 93) that promotes prion formation and is crucial for the maintenance of different prion phenotypes. On the other hand, the middle domain (M-domain) of Sup35 is highly charged (with lysine and glutamine) (Helsen and Glover 2012). This region is intrinsically disordered in purified samples of the NM protein yet is required for the inheritance of the prion from mother to daughter cells (Liu, Sondheimer and Lindquist 2002). The M domain adopts an ordered conformation when fibers are assembled inside cellular lysates, presumably through stabilizing interactions with molecular chaperones (Frederick *et al.*^2015^). These two domains are necessary and sufficient for the prion activity of Sup35 and are referred to collectively as NM (Li and Lindquist 2000). The C-terminal region (residues 254 - 685) performs the translation termination function and is completely extraneous to prion behavior per se.

Electron microscopy (EM) can provide important information about the structural diversity of amyloids. Indeed, mass-per-length (mpl) measurements by scanning transmission electron microscopy (STEM) have contributed important constraints for determination of the molecular structures of a variety of amyloids (Diaz-Avalos *et al.*^2005^; Komatsu *et al.*^2010^). For the yeast prion protein, fiber morphologies and mpl measurements have been reported for full length as well as several truncations of Sup35 for de novo fibers assembled under conditions that favor formation of fibrils that confer the strong prion phenotype (Baxa *et al.*^2011^). Variant-specific morphologies and mpl have been reported in detail for amyloids formed by the N-terminal 61 residues of Sup35 fused with GFP, N (1–61)-GFP (Diaz-Avalos *et al.*^2005^). However, studies on the structural polymorphisms that lie at the heart of the yeast prion strains and their correlation with mpl have not yet been fully explored. Additionally, prior work was done on in vitro formed fibers, which may not represent the biologically active structure.

In this study, we used EM to characterize the NM protein templated into the prion form by cellular lysates of yeast manifesting either the strong or weak [*PSI*^+^] biological phenotypes. Introduction of these lysate templated preparations of NM fibrils back into [*psi*^−^] cells conferred pure prion phenotypes (Dong *et al.*^2010^; Frederick *et al.*^2014^), rather than mixtures as is seen with *de novo* material. Using this approach, we investigated the mpl and other structural polymorphism of lysate-templated NM fibrils that can confer either pure strong or pure weak prion phenotypes.

## MATERIALS AND METHODS

### Sample preparation

Recombinant prion domain (NM) of the yeast prion protein Sup35 was purified as described (Serio *et al.*^1999^). Pure prion stains were obtained by templating denatured NM into the amyloid form using lysates from yeast harboring either the strong or weak prion phenotype through several rounds of amplification as previously described (Dong *et al.*^2010^; Frederick *et al.*^2014^). Isotopically enriched NM was obtained by expressing NM in BL21 *E. coli* cells in the presence of minimal media enriched with 1g/L of ^15^NH_4_Cl and 2g/L D-glucose ^13^C_6_ (Cambridge isotope labs) (Frederick *et al.*^2014^). Segmentally isotopically labeled versions of the NM protein were produced biosynthetically using split inteins to ligate the isotopically labeled and unlabeled segments. Inteins are segments of a protein that are able to excise them-selves from a polypeptide chain and join the remaining portions with a peptide bond. The genes for the naturally split Npu DnaE Intein and the prion domain of the yeast prion protein NM, were codon-optimized for bacterial expression by GeneScript. Both constructs were made by overlap-PCR and ligated into pET vectors. Construct1 contained the first 32 amino acids of NM followed by first 102 amino acids of Npu DnaE sequence and was cloned into the pET 28a vector (Invitrogen) at the Nde1 and Xho1 sites, using the N-terminal hexahistidine tag and thrombin cleavage site encoded in the vector. Construct2 contained the last 36 amino acid of the Npu DnaE intein, a cysteine and residues 33–253 of NM, and was cloned into the NdeI and XhoI sites of pET-22b vector (Invitrogen) using the C-terminal hexahistidine tag encoded by the vector. Expression of proteins split intein ligation reaction and construction of segmentally labeled NM proteins were done as previously described. Ligated protein was purified using affinity chromatography based on the presence of both an N and C terminal affinity tag to separate full length molecules from truncated versions, as previously described (Frederick *et al.*^2017^).

### Fibril preparation and solid state NMR spectroscopy

Purified NM was templated into the amyloid conformation by using amyloid seed from lysate-templated polymerization reactions for both strong and strong fibrils as previously described (Frederick *et al.*^2014^). NM fibrils samples for EM were assembled in 10 mM HEPES at pH 7.2 to eliminate salt that can be potentially confounding for mpl measurement. Fiber samples for NMR analysis were assembled in 5 mM potassium phosphate pH 7.2 and 150 mM NaCl. Fiber sample of segmentally labeled proteins that contain a Cys residue were assembled in the same buffer that also contained 1 mM TCEP (Frederick *et al.*^2017^). Solid-state magic angle spinning (MAS) NMR spectra were collected with a spectrometer operating at 700 MHz ^1^H Larmor frequency with 12.5 kHz MAS and an estimated sample temperature of 10°C as previously described (Frederick *et al.*^2014^). ^15^N-^13^C correlations were recorded with TEDOR dipolar recoupling (Jaroniec, Filip and Griffin 2002) with a mixing period of 1.6 ms and a recycle delay of 3 seconds. Spectra were processed in NMR pipe (Delaglio *et al.*^1995^) and analyzed using Sparky.

### STEM Image acquisition and Data Processing

Specimens for STEM mpl were prepared on titanium grids coated first with thick holey carbon (∼4 micron holes) followed by thin (∼3 nm thick) carbon. The thin carbon was prepared by vacuum evaporation onto freshly cleaved single-crystal rock salt in an ion-pumped bell jar. The carbon was floated on distilled water and the holey-film grid was applied to the exposed surface. The grid was picked up with tweezers, retaining a drop of liquid. The hanging drop was partially wicked away and 5 μLs of TMV (tobacco mosaic virus) injected into the remainder of the hanging drop. This was followed by 3 drops of ammonium acetate (20 mM, pH 7) and one drop of specimen solution (∼30 μg/mL). After 1 minute the grid was washed 10 times with ammonium acetate (volatile buffer), blotted with filter paper and frozen in liquid nitrogen slush. Grids were transferred to an ion-pumped freeze dryer, freeze dried overnight by gradually warming from −160°C to −90°C and transferred under vacuum to the STEM cold stage operating at −160°C. Specimens for staining were prepared in the same way except for a final wash with uranyl acetate: 2% for negative stain, 10^−5^ M for positive staining.

The STEM was operated at 40 keV with a beam focused to <0.3 nm. Measurements were made with a pixel spacing of 1 or 2 nm in a 512 × 512 square array. Scattered electrons were collected on an array of detectors: 0–15 mRadian scattering angle (Bright Field), 15–40 mR (Small Angle) and 40–200 mR (Large Angle) elastic scattering. All 3 signals were recorded digitally. Since the beam current from the cold field emission gun fluctuates over time, signals were normalized by the sum of all detectors.

Mass measurements were performed off-line using a custom program, PCMass32 (ftp.stem.bnl.gov/pub/pcmass32). Since the STEM measures total mass in the path of the beam, background subtraction is critical, especially for thin extended structures. PCMass does this by masking particles above a threshold, masking an additional radius, averaging remaining pixels in a 4 × 4 grid and interpolating to give background at intermediate points. Since the STEM detectors have quantum efficiency, measurement of background signal and standard deviation allows computation of dose (typically 10–20 el/Å^2^). Tobacco mosaic virus (TMV) was included as an internal control in all specimens to check STEM M/L calibration, quality of shape preservation and absence of salt. Any regions dried in air prior to freezing show accumulation of salt along the edge of the TMV in a characteristic distortion of the cylindrical profile. PCMass locates objects and traces filaments, tabulating segment Mass, M/L or mass per unit area. The program can perform statistical analysis and supplies all data in a spreadsheet.

## RESULTS

### Fibril morphology

To ensure that fibrils were both biologically homogenous and of clear relevance to the *in vivo* prion state, we seeded polymerization with lysates of cells carrying phenotypically strong or weak prion elements. For strong prions, polymerization was performed at 4°C, a condition that favors assembly of amyloids conferring the strong prion phenotype (strong NM fibrils). For weak prion elements, polymerization reactions were seeded at 37°C, which favors assembly of amyloids conferring a weak prion phenotype (weak NM fibrils). When non-prion yeast cells were infected with these in vitro assembled amyloids, they produced specific and pure prion phenotypes (Dong *et al.*^2010^; Frederick *et al.*^2014^).

**Figure 1.**
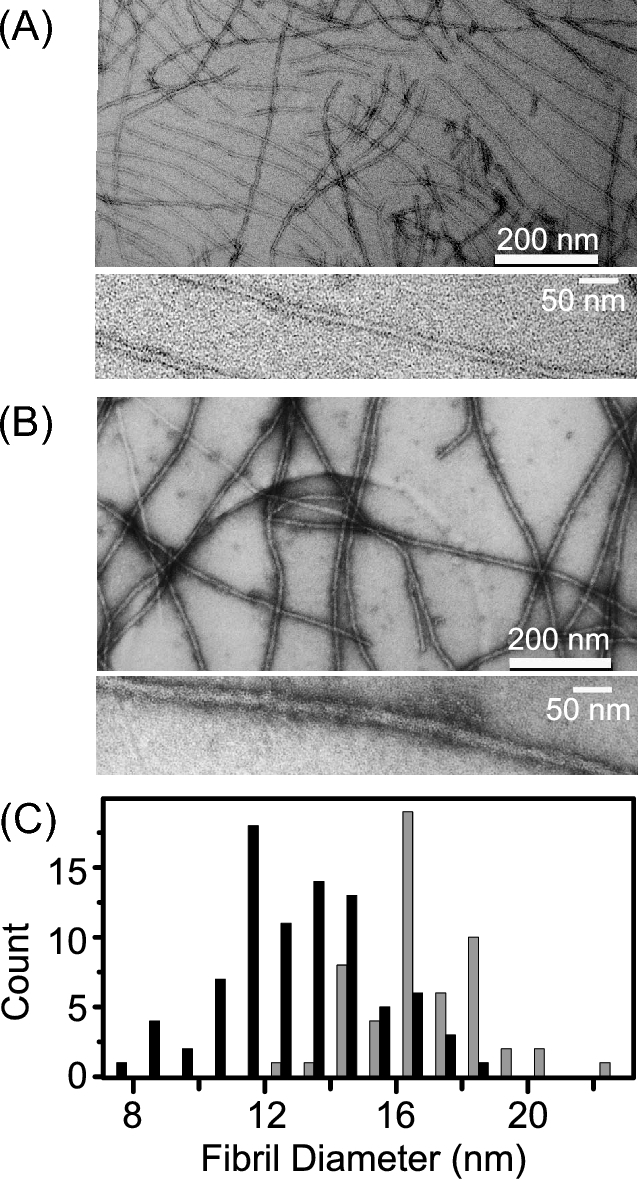
Morphological diversity was observed for NM fibrils that embody pure prion phenotypes. (**A**) Strong and (**B**) weak NM fibrils at low (top) and high (bottom) magnification. Polymerization of this sample was seeded by lysates of cells carrying phenotypically strong or weak prion elements, respectively. Fibril assembly was performed under conditions that favor assembly of amyloids that confer the ‘strong’ or ‘weak’ prion phenotype. (**C**), Histogram representing the average diameter of strong (black) and weak (grey) NM fibrils. Strong NM fibrils have an average diameter of 11.5 nm while weak NM fibrils have an average diameter of 16 nm.

Lysate templated fibers manifested a range of fibril morphologies by negative staining EM. For samples from both variants, we found two major populations defined by their fibril diameter, referred to as ‘thick’ and ‘thin’ fibrils, as shown in Fig. 1A and B. Thin fibrils had a diameter of 8–14 nm, while the thick fibrils had a diameter of 12–20 nm (Fig. 1C). Although both types of fibril were observed in strong and weak NM fibril samples, they comprised different percentage of population. The majority of the strong NM fibrils were thin, while the majority of the weak NM fibrils were thick. Strong NM fibrils had an average of diameter of 11.5 nm, smaller than that of weak NM fibrils, which had an average diameter of 16 nm (Fig. 1C). Although we repeatedly observed fibrils with different width distribution, such measurements are notoriously sensitive to variations in the negative staining technique. Thus, we visualized unstained amyloid fibrils using dark-field STEM.

Similar mixtures of thin and thick morphologies were observed. In the dark-field STEM, the image is generated by contrasting the specimen scattering directly, with no complication from staining. In the absence of staining, both ‘thin’ and ‘thick’ fibrils were observed in samples of both strong and weak NM fibrils, albeit with clearly different populations. In the samples of strong prion fibrils, about 80% of the fibrils (305 out of 381 fibrils) were thin, while the remaining 20% were thick. In contrast, only about 32% of the fibrils (106 out 330 fibrils) were thin in samples carrying weak prion state, while the majority of the fibrils (68%) were thick.

### Mass per length (mpl) measurements for different fibril types

**Figure 2.**
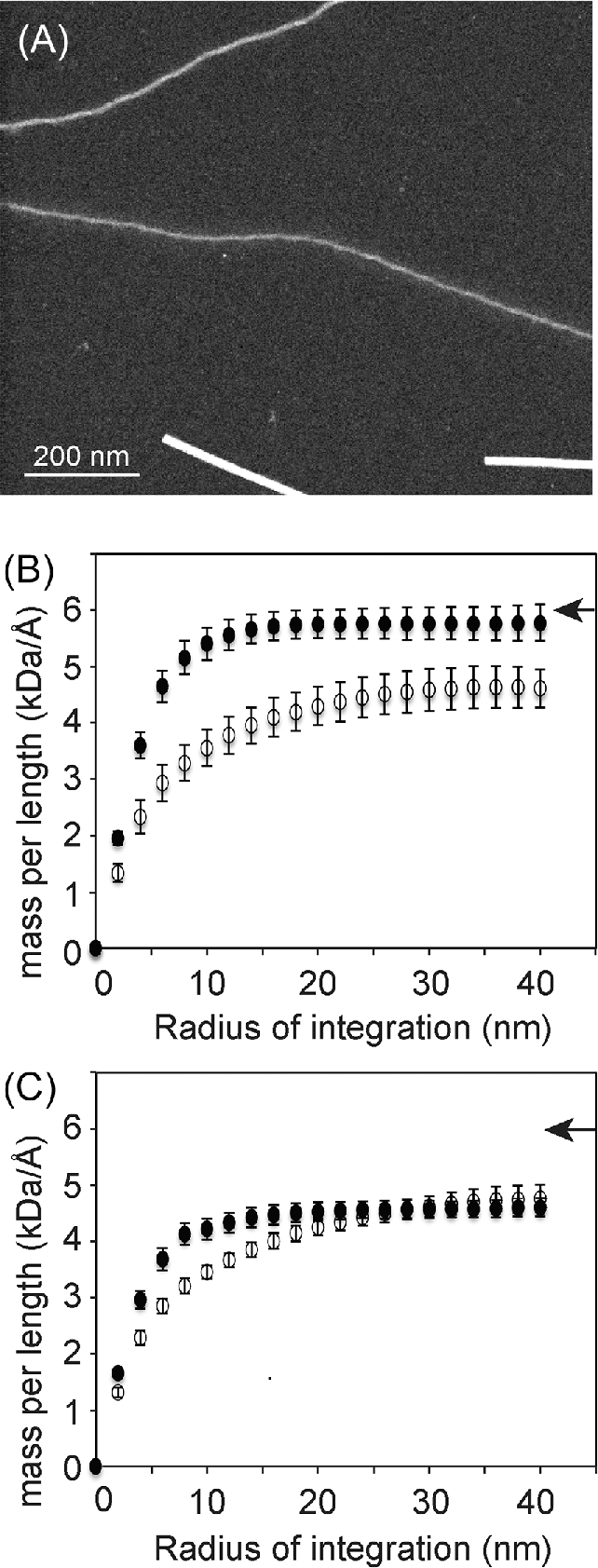
(**A**) Dark field STEM image of unstained NM fibrils contained mixtures of thick NM fibrils (top) and thin NM fibrils (middle). TMV is shown as a standard (bottom), which is saturated in this image due to contrast adjustments to enable visualization of thin filaments. Plots of the mpl versus the radius of integration for (**B**) thick (closed circles) and thin (open circles) forms of strong NM fibrils and (**C**) thick (closed circles) and thin (open circles) forms of weak NM fibrils. Arrowheads indicate the mpl value that corresponds to one NM monomer (28.5 kDa) per axial cross beta distance (0.47 nm).

Dark-field STEM of the unstained samples not only provided an alternative imaging method, but more importantly, allowed the direct measurement of mpl for different fibril types (Fig. 2A). The apparent mpl for the dominant (80%) thin form of strong NM fibrils increases slowly as the integration extends longer distances from the fibril center. While half of the mpl is accounted for within 4 nm of the fibril center, this value continues to increase slowly towards 4.6 ± 0.4 kDa/Å as the radius of integration is increased to 30 nm. This is in contrast to the less common (20%) thick forms of strong NM fibrils, where half of the mpl is accounted for within 3 nm of the fibril center and reaches its final value of 5.8 ± 0.3 kDa/Å with a radius of integration of 16 nm, half that of the thin fibril form (Fig. 2B). Using 28.5 kDa as the molecular weight of the NM protein and 0.47 nm for the cross beta spacing, the number of protein subunits per axial length is around 0.75 for the thin form of these strong NM fibers, which are the majority species, and 0.95 for the thick form. The situation is similar for the weak NM fibrils, except the final mpl measurement of neither thick nor thin fibrils approaches one monomer per cross-beta spacing. Rather both are similar to the thin strong fibers and have mpl near 0.75 monomers per cross beta spacing. The mpl of the less common (32%) thin form of weak NM fibrils increases slowly as the radius of integration extends further from the fibril center. Half of the mpl for the thin weak NM fibrils is accounted for within 4 nm of the fibril center and the mpl continues to increase to a final value of 4.7 ± 0.2 kDa/Å over the next 34 nm. In contrast, half of the mpl of the dominant (68%) thick form of the weak NM fibrils is accounted for within 3 nm of the fibrils center and the mpl reaches its final value of 4.6 ± 0.2 kDa/Å with a radius of integration of 20 nm (Fig. 2C). Relative to the thick forms, the thin forms of NM fibrils had a smaller fibril core with the protein extending further from the center.

### The thick and thin forms of strong and weak NM fibrils have distinct atomic structures

**Figure 3.**
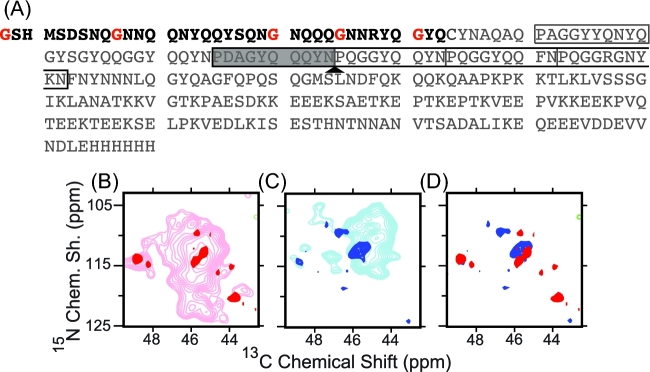
(**A**) Sequence of NM. The segmentally isotopically labeled region is in black and the unlabeled region is in gray. Isotopically labeled glycine residues are highlighted in red. The five imperfect oligopeptide repeats in the wild type sequence are indicated by boxes. The repeats in the black box are deleted in the RΔ2–5 construct. Two copies of the shaded repeat are inserted at the arrow in the R2E2 construct. Overlay of the carbon–nitrogen correlation spectra for (**B)** uniformly isotopically labeled weak NM fibrils (pink) and segmentally isotopically labeled weak NM fibrils (red) (**C**) uniformly isotopically labeled strong NM fibrils (light blue) and segmentally isotopically labeled strong NM fibrils (dark blue). The overlay of (**D**) segmentally isotopically labeled strong (blue) and weak (red) NM fibrils illustrates that the glycine residues in these fibers experience distinct chemical environments, which suggested that thick and thin fibrils of both the strong and weak NM fibril forms had distinct structures.

To determine if the thick and thin fibers have same structure at the atomic level, we collected the MAS NMR spectra of isotopically labeled NM templated into their prion conformation from cellular lysates. MAS NMR experiments report only on regions with high molecular order; regions of the protein that undergo dynamic motions are not visible in these experiments. NMR spectra of uniformly isotopically labeled weak and strong NM fibrils revealed the presence of more rigid structure of the N domain in weak fibrils than strong fibrils, in agreement to prior work (Frederick *et al.*^2014^). However, chemical shift degeneracy precluded a detailed analysis. Thus, to determine if the weak and strong NM fibrils share a molecular structure, we isotopically labeled only a segment of the NM protein (Frederick *et al.*^2017^) (Fig. 3A). While most regions of the carbon–nitrogen correlation spectra (TEDOR) were still too crowded for detailed analysis (data not shown), the glycine region was sufficiently resolved. The glycine spectra of the weak NM fibrils (Fig. 3B) had four major peaks and four minor peaks, all with different chemical shifts. Prion fibrils are heterogeneously dynamic protein assemblies that experience motion over a broad range of timescales (Frederick *et al.*^2014^) and dynamic motions can complicate the direct interpretation of peak intensities as populations. Yet, for the more biophysically robust NM fibers that confer the weak prion phenotype, the intensities of the four major peaks were 3.3 times greater (24 ± 10 a.u.) on average than the four minor peaks (7 ± 0.8 a.u.). The relatively equal peak intensities across the four major and four minor peaks near the ratios observed for thick and thin weak NM fibrils suggest that these peaks report on these two distinct fibril morphologies. The different chemical shifts suggest different underlying molecular structures and the narrow lines in the spectra indicate high molecular order (0.8 ± 0.4 ppm). Likewise, the glycine spectra of the strong NM fibrils had three major and three minor peaks, all with different chemical shifts. The overlay of the spectra for the weak and strong NM fibrils showed that all of the glycine peaks had distinct chemical shifts. Thus, the NMR data suggest that there are four distinct conformations for the amyloid core, one for thick weak NM fibrils, one for thin weak NM fibrils, one for thick strong NM fibrils and one for thin strong NM fibrils.

### The M domain contributes to the structural polymorphism

The morphological imaging, mpl measurement and solid state NMR spectroscopy showed that the N domain of NM can assemble into several fibril forms even under conditions in which the resulting fibrils induce distinct and biologically pure prion phenotypes as shown above. To determine how the M domain contributed to the observed structural polymorphism, we sought to specifically visualize the M domain using dark field STEM. In the negatively stained EM, high concentrations of uranyl acetate solution (2%) were used to stain the sample, resulting electron dense background and electron lucent fibrils. Since uranyl acetate can chelate carboxyl groups strongly, we also used 0.02% uranyl acetate to stain the sample (positive staining) and imaged the samples by dark-field STEM. At such low concentration, uranyl acetate will preferentially interact with the M domain, which is highly populated with glutamate residues (23 glutamate residues in the M domain; zero in the N domain). Therefore, positive staining of NM fibrils by uranyl acetate may allow us to differentiate N and M domain and investigate their individual contribution to the structural polymorphism.

**Figure 4.**
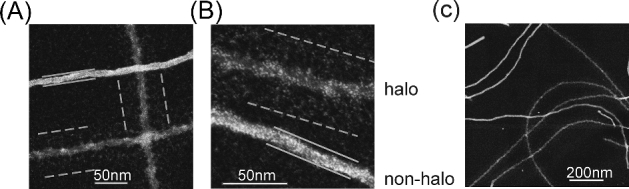
The M domain contributes to the structural polymorphism. (**A**) and (**B**), Thin and thick fibrils show very different staining profiles. Dashed lines show the range of positive staining by heavy atom clusters around the fibril core in thin fibrils, described as a ‘halo’. Solid lines indicate a compact fibril core in thick fibrils representing non-halo fibril. (**C**), Low magnification image showing that the boundaries of thick fibrils are well defined but boundaries of thin fibrils are more spread out.

Thin and thick fibrils had very different positive staining profiles (Fig. 4A). The uranyl acetate accumulated on the main body of the thick fibrils, resulting in thick bright morphology. The staining edge of the thick fibrils is well defined, as indicated by the solid lines in Fig. 4A. The main body of the thin fibrils is less bright with significantly more density of staining clusters extending on both sides of the fibril, forming a halo around the fibril core. In this case, the fibril edge is much less well defined with a much wider staining diameter (as indicated by the dashed line in Fig. 4A). Thus the thin fibrils had a ‘halo’ and the thick fibrils do not. When referring to this difference, we will refer to halo and non-halo fibrils. Since the uranyl acetate has strong tendency to bind the glutamate in the M domain, this result indicated that the M domain had different arrangements in the halo and non-halo fibrils. In the thin fibrils, the M domain, being flexible, was extended outside the amyloid core formed by the N domain. In the thick fibrils, the M domain and the N domain are in close proximity; the M domain was either folded together with the N domain to form the amyloid core or alternatively was still outside the core but collapsed onto the surface.

Strikingly, we also noticed that strong NM fibrils, which have a majority of thin fibrils, tend to align themselves with a uniform interfibril spacing (Fig. 1A up and 4C) (Baxa *et al.*^2003^). This equi-spaced arrangement may be a result of the electrostatic repulsion of the highly charged M domain being extended and on the outside of the amyloid core. In contrast, such an ordered arrangement was seldom observed in the weak NM fibril samples, consistent with a more compact structure of the M domain in the thick fibrils.

To determine if thick non-halo fibrils are a mature form of thin halo fibrils that is formed by collapsing the M domain over time, we examined the samples after different incubation times: 2 hours, 72 hours and 1-week. Both thin halo and thick non-halo fibrils were observed as early as 2 hours and the relative population of the thin and thick fibrils didn’t change over time, suggesting that the thick non-halo fibrils form once the assembly process is initiated. Thus, the thick non-halo fibrils do not represent a maturation product of the thin halo fibrils. This underlies the results of the NMR spectroscopy and provides further evidence of non-convertible distinct structures.

### The mpl measurement for NM proteins with repeat deletion or expansion in the N domain

Multiple lines of evidence indicate that the residues of the N domain form the amyloid core, yet it is still not clear how monomers are arranged into fibers. Because the repeat region of the N domain is essential for the maintenance of different prion phenotypes, despite evidence that it is not always directly involved in protected cross-beta strands, we applied dark-field STEM to fibrils formed from NM protein carrying a deletion of repeats two through five (RΔ2–5), or carrying two repeat expansion (R2E2) (Fig. 3A). Samples were seeded by strong prion seeds at 4°C. As previously shown (Castro *et al.*^2011^), both RΔ2–5 and R2E2 fibrils assembled under such condition induced uniform strong prion phenotypes when assayed by protein-only transformation.

**Figure 5.**
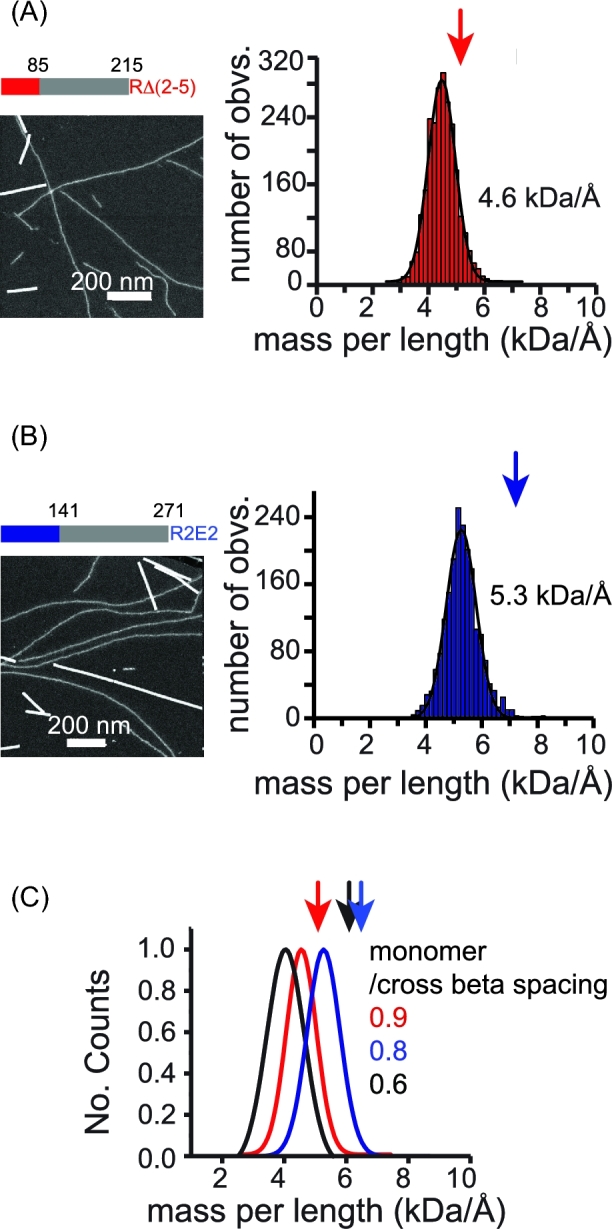
(**A**) RΔ2–5 fibrils, which have a deletion in the repeat region of the N domain of NM (colored bar) and (**B**) R2E2 fibrils, which have an expansion in the repeat reagion of the N domain (colored bar) that embody strong prion phenotype are visualized by STEM (***Left***) and the corresponding histograms of mpl measurements (***Right***). **(C)** Comparison of the Gaussian fits for NM (black), RΔ2–5 (red) and R2E2 (blue) fibrils that embody strong prion phenotype. The arrowheads indicate the mpl value for each protein if one monomer expands 4.7 Å cross beta axial distance, based on protein molecular weights of 4.6 and 5.3 kDa, respectively. The RΔ2–5 and R2E2 constructs are described in Fig 3a.

To our surprise, strong NM fibrils made from both RΔ2–5 and R2E2 appeared as relatively uniform non-halo fibrils (Fig. 5A and B). These preparations were much more homogenous than those of strong wild type NM fibrils (which consist of 20% non-halo fibrils and 80% halo-fibrils). Thus, although the wild type and both repeat region mutants of NM confer the strong phenotype, the conformation of the M domain is biased towards a compacted (non-halo) configuration by modification of the repeat region. This suggests that the region that encodes prion phenotype strength is at the extreme N terminal region of the fiber, consistent with the conformation and size of the amyloid core being the driver of prion strain determination. The strong RΔ2–5 fibrils have an average mpl of 4.6 kDa/Å (0.9 monomer/cross-beta sheet spacing), while strong R2E2 fibrils have a mpl of 5.3 kDa/Å (0.8 monomer/cross-beta sheet spacing).

**Figure 6.**
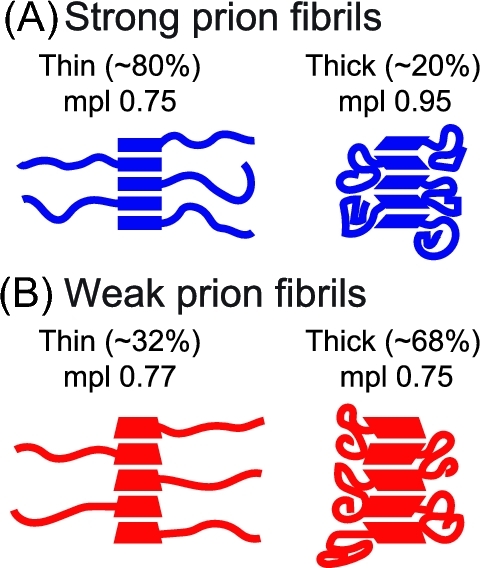
Model of the four different fibril forms. Quadrilaterals represent the N domain of NM, which exists in four distinct conformations. Curved lines represent the M domain, which is either extended in the thin ‘halo’ fibrils or compacted in the thick ‘non-halo’ fibrils. (**A**) Strong prion fibrils are mostly ‘thin’ and have an mpl of 0.75 NM monomers per cross beta spacing. (**B**) Weak prion fibrils are mostly ‘thick’ and also have an mpl of 0.75 monomers per cross beta spacing, although the underlying molecular organization of the N domain is distinct.

## DISCUSSION

Yeast prions represent a paradigm-shifting, protein-based mechanism for the inheritance of biological phenotypes. The strong and weak prion strains of Sup35 confer distinct heritable phenotypes that are based on different self-templating amyloid conformations of the same protein (Toyama *et al.*^2007^). Despite intense study, we still have only a rudimentary understanding of the structural differences that underpin the inheritance of distinct phenotypes. Previous work has established that the N-terminal domain forms the amyloid core of de novo NM fibers and that the core of fibers formed at 4°C is shorter than that of fibers formed at higher temperatures(Krishnan and Lindquist 2005; Toyama *et al.*^2007^; Frederick *et al.*^2014^). Fibers formed at 4°C are biased toward structures that confer strong prion phenotypes *in vivo*, while fibers formed at higher temperatures are biased toward structures that confer weaker phenotypes. Spontaneously assembling fibers always contain some mixture of forms as evidence by a mixture of phenotypes in protein transformations from de novo assembled fibers (Hess, Lindquist and Scheibel 2007). In this study, we ensured the biological purity of specimen using well-controlled native polymerization condition in cellular lysates and confirming the prion status by protein-only transformation assay (Tanaka *et al.*^2004^). Thus, to our surprise, for both prion forms, we observed two distinct fiber morphologies, thick and thin. Using MAS NMR spectroscopy, we establish that the region of the prion that drives heritable, strain-specific differences in prion polymerization (the N-terminal amyloid domain) can assume at least four distinct conformations. Using segmentally isotopically labeled NM molecules (Frederick *et al.*^2017^), we found that these thin and thick fibrils of both the strong and weak variants were all structurally distinct.

The molecular structure of NM fibrils has not been determined, but two general models have been proposed, a beta-pleated sheet model (Shewmaker, Wickner, and Tycko 2006) and a beta-helix model (Krishnan and Lindquist 2005). In the beta-pleated sheet model, each monomer is stacked on top of the next along its entire length, one monomer per rung and each amino acid on top of the same amino acid in the adjacent monomers. With such arrangement, one monomer per cross-beta sheet spacing is expected. The mpl measurement therefore provides an important constraint for determination of the structure of amyloid fibrils. The mpl has been previously measured for various preparations of NM fibrils. One of the studies used a tilted-beam transmission electron microscopy (TEM) (Diaz-Avalos *et al.*^2005^), a technique generally not recommended for mpl measurement of unstained specimens. This technique requires significantly higher electron dose compared to dark field STEM for the same counting statistics, raising concerns about damaging biological samples. Moreover, electron dense phosphate buffer gave rise to apparent salt contamination in the study. In another study, Sup35p was spontaneously assembled into fibrils capable of conferring the strong prion phenotype and measured using dark-field STEM. Using full length Sup35p and several truncations, this work found that the N domain formed the amyloid core, the M domain was extended and the C terminal domain was arrayed around the fibers, suggesting that the domain could retain translation termination activity (Baxa *et al.*^2011^). In this work, all the Sup35 fibrils resulted in a single fiber type, all of which had a mpl of one monomer/cross-beta sheet spacing (Baxa *et al.*^2011^). However, because spontaneously assembled fibers are known to be somewhat heterogeneous, it was surprising that a single mass population was observed.

Using lysate-templated samples of strong and weak NM fibrils, we found that NM fibrils embodying different prion phenotypes have distinct mpl. Moreover, the mpl results on different NM mutants indicated that the difference in mpl is a combination of differential packing of both the N as well as the M region. However, the ability of NM with a truncated or modified M domain to assemble into fibrils with different mpl that can support both the strong or the weak prion states, suggests that the differential packing of the N domain, but not the M domain, is the most likely the determinant for the existence of distinctive prion phenotype. A recent study on the de novo assembly process for NM fibers revealed an additional fiber form that sequesters the repeat region of NM into amyloid fibers, leaving the N terminal region disordered (Ohhashi *et al.*^2018^). Fibers samples containing a mixture of this alternative fibril form with the fibril form that sequesters the N terminal most region were suggested to responsible for the observation that NM fibrils have interacting regions between the N terminal most regions (heads) and the repeat regions (tails) (Krishnan and Lindquist 2005). While we observe a mixture of fibril forms in our lysate templated samples, all of them sequester the N terminal most region in the amyloid fiber; we observed signals from the first 32 amino acids by solid state NMR in all of the fibril forms so this region must be rigid in all of these variants. Finally, of all of the NM fibrils observed, the mpl varied from 0.75 to 0.95 monomers per cross-beta sheet spacing. Only one fibril form, the thick form of the strong NM fibrils, had a mpl that corresponded to ∼1 monomer per cross-beta sheet spacing (Fig. 6). Thus, it seems unlikely that the monomers in lysate-templated fibrils adopt parallel in register beta pleated sheets, in line with a recent solid state NMR study on lysate-templated strong NM fibrils (Frederick *et al.*^2017^).

Unlike the N domain, considerable confusion exists about the fundamental nature of the M domain of the NM prion. It has been alternatively reported to be flexible (Krishnan and Lindquist 2005; Toyama *et al.*^2007^; Frederick *et al.*^2014^) or to be primarily in a rigid parallel-in-register amyloid core (Shewmaker, Wickner and Tycko 2006; Shewmaker *et al.*^2009^). We do not know what accounts for these discrepancies. They might reflect the use of fibers assembled de novo, which contain heterogeneous mixtures of multiple fiber forms (Hess, Lindquist and Scheibel 2007; Toyama *et al.*^2007^; Shewmaker *et al.*^2009^) or simply the liabilities in the interpretation of experimental outputs from the distinct techniques used. Indeed, most prior work reported upon characteristics of the entire ensemble of fibrils in the sample. In contrast, EM allows for study of individual fibers (albeit with consequent sampling issues) and revealed that lysate-templated samples of both strong and weak NM fibrils were a mixture of two major populations defined by their fibril diameter. When we visualized the M domain by dark field STEM, we found that the M domain in thin fibers extended away from the fibril core while in thick fibrils the M domain was in close proximity to the N domain. Thin and thick fibril forms did not interconvert. Interestingly, hints of such standing heterogeneity were observed over a decade ago by hydrogen-deuterium exchange; some sites had fast initial exchange rates and then remain stable at an intermediate level for the remainder of the time course (Toyama *et al.*^2007^). Such sites are more common in the de novo fibers prepared at 37°C than for those prepared at 4°C and include a fifth of the 55 sites measured in the M domain.

The mixed morphologies of these fibers, which were templated from cellular lysates, raise questions about the fidelity of in vitro amyloid amplification reactions from *in vivo* sources. It is formally possible that these yeast prion variants result from a propagated mixture of two distinct amyloid conformations. Indeed, these preparations faithfully confer a single prion phenotype in lysate conformations. Yet, the transformation efficiency is significantly lower than obtained from preparations of de novo prepared fibers, raising the possibility that the lower transformation efficiency is a result of only one of the two conformations being the one that is propagated *in vivo*. This is reminiscent of some of the barriers encountered when attempting to amplify amyloid aggregates of the proteins that are involved in neurodegenerative diseases. The conformations that are biophysically favored for amplification *in vitro* may not be those that are propagated *in vivo*. For example, Sup35 was recently found to form phase-separated droplets in response to cellular stress (Franzmann *et al.*^2018^). Differences in these condensates, perhaps as a result of different cellular stressors, are likely difficult to recapitulate in vitro yet may result in different fibril structures. Moreover, interactions with cellular constituents may be key in conformational selection and prion propagation. Indeed, there are dozens of cellular factors that enhance as well as compromise prion propagation and inheritance. This was perhaps most recently illustrated by a structural study of strong NM fibrils assembled at endogenous levels in cellular lysates. The M domain, which is largely intrinsically disordered in purified samples underwent a dramatic secondary structural rearrangement, presumably as a result of interactions with molecular chaperones (Frederick *et al.*^2015^). That an intrinsically disordered region becomes structured in a cellular environment is an extreme example of how different environments can modulate the energetic landscape for protein folding to favor a different protein conformation. Such forces are likely also at play for the amyloid-forming region of the yeast prion protein. To truly know which conformations are those responsible for these non-Mendalian protein based elements of inheritance, we will need to determine protein conformations *in situ*.
